# Deeply divergent archaic mitochondrial genome provides lower time boundary for African gene flow into Neanderthals

**DOI:** 10.1038/ncomms16046

**Published:** 2017-07-04

**Authors:** Cosimo Posth, Christoph Wißing, Keiko Kitagawa, Luca Pagani, Laura van Holstein, Fernando Racimo, Kurt Wehrberger, Nicholas J. Conard, Claus Joachim Kind, Hervé Bocherens, Johannes Krause

**Affiliations:** 1 Institute for Archaeological Sciences, University of Tübingen, Rümelin Strasse 23, Tübingen 72070, Germany; 2 Max Planck Institute for the Science of Human History, Khalaische Strasse 10, Jena 07745, Germany; 3 Department of Geosciences, Biogeology, University of Tübingen, Hölderlin Strasse 12, Tübingen 72074, Germany; 4 Department of Prehistory, National Museum of Natural History, UMR 7194 CNRS, 1 rue René Panhard, Paris 75013, France; 5 Estonian Biocentre, Riia 23b, Tartu 51010, Estonia; 6 Department of Biology, University of Padova, Via Ugo Bassi 58/B, Padova 35121, Italy; 7 Department of Archaeology and Anthropology, University of Cambridge, Fitzwilliam Street, Cambridge CB2 1QH, UK; 8 New York Genome Center, 101 Avenue of the Americas, New York, New York 10013, USA; 9 Ulmer Museum, Marktplatz 9, Ulm 89073, Germany; 10 Department of Early Prehistory and Quaternary Ecology, University of Tübingen, Schloss Hohentübingen, Tübingen 72070, Germany; 11 State Office for Cultural Heritage Baden-Württemberg, Berliner Strasse 12, Esslingen 73728, Germany; 12 Senckenberg Centre for Human Evolution and Palaeoenvironment, University of Tübingen, Hölderlin Strasse 12, Tübingen 72074, Germany

## Abstract

Ancient DNA is revealing new insights into the genetic relationship between Pleistocene hominins and modern humans. Nuclear DNA indicated Neanderthals as a sister group of Denisovans after diverging from modern humans. However, the closer affinity of the Neanderthal mitochondrial DNA (mtDNA) to modern humans than Denisovans has recently been suggested as the result of gene flow from an African source into Neanderthals before 100,000 years ago. Here we report the complete mtDNA of an archaic femur from the Hohlenstein–Stadel (HST) cave in southwestern Germany. HST carries the deepest divergent mtDNA lineage that splits from other Neanderthals ∼270,000 years ago, providing a lower boundary for the time of the putative mtDNA introgression event. We demonstrate that a complete Neanderthal mtDNA replacement is feasible over this time interval even with minimal hominin introgression. The highly divergent HST branch is indicative of greater mtDNA diversity during the Middle Pleistocene than in later periods.

## Abstract (web summary)

Ancient DNA keeps expanding our understanding of complex genetic relationships between Pleistocene hominins. Here, Posth and colleagues analyse the mitochondrial genome of an archaic human that diverged from other Neanderthals ∼270,000 years ago, providing the minimum age for an African introgression into Neanderthals.

In recent years, an increasing number of mitochondrial DNA (mtDNA) and nuclear genome (nDNA) data from archaic human remains have reshaped the understanding of evolutionary relationships among various hominin groups. Mitochondrial genomes provided evidence for at least two distinct mtDNA branches associated with Neanderthals and Denisovans, respectively, suggesting a sister group relationship between modern humans and Neanderthals with Denisovans as a basal mtDNA outgroup^1^,^2^,^3^,^4^. However, nDNA data revealed that Neanderthal and Denisovan populations separated only after their divergence from the lineage leading to modern humans^2^,^5^,^6^,^7^,^8^.

The estimate for the population split time between the two archaic hominin groups and modern humans was calculated to 765,000–550,000 years ago (765–550 ka) based on a recent estimate of the genome-wide human mutation rate^5^. Furthermore, analyses of Y-chromosome data from a male Neanderthal returned an age of 806–447 ka for the divergence of Neanderthal and modern human Y-chromosome lineages^9^. These time intervals largely overlap, suggesting that the Neanderthal Y chromosome differentiated through the population split from the most recent common ancestor (MRCA) of modern humans and Neanderthals. In contrast, the corresponding divergence time for mtDNA has been dated to ∼400 ka (95% highest posterior density (HPD), 498–295 ka)^10^,^11^ and was thus found to be considerably younger compared to the time estimates obtained from autosomal and Y-chromosome data.

In addition, nDNA analyses of the Middle Pleistocene hominins from the Sima de los Huesos site in northern Spain confirmed their closer affinity to the Neanderthal lineage^8^, suggesting that at least by ∼430 ka, Neanderthals and Denisovans had already diverged (Fig. 1d). However, in contrast to genome-wide data, the Sima de los Huesos mtDNA was found to branch off with the deeply divergent Denisovan mtDNA lineage^3^. The phylogenetic discrepancies could be reconciled if the mtDNA of early Neanderthals was indeed Denisovan-like and was subsequently replaced by a more derived mtDNA lineage. Therefore, a genetic introgression event from African hominins into the early Neanderthal population that gave rise to the ‘Late Pleistocene’ Neanderthal mtDNA lineage has been proposed^8^. This event must have occurred after archaic and modern human populations diverged. However, the exact timing of the proposed gene flow is unknown and merely based on possible archaeological evidence for contacts between African and Eurasian populations^8^.

While genomic evidence showed that gene flow between lineages as divergent as modern humans and Neanderthals took place^12^,^13^ in both directions^14^, it is unclear whether such small-scale phenomena were sufficient to explain the complete replacement of the initial Neanderthal mtDNA pool (found in Sima de los Huesos) by a Middle Pleistocene human lineage from Africa. Moreover, the temporal placement of such admixture event into Neanderthal populations is still under debate, partly due to the limited availability of additional archaic DNA. Therefore, an assessment of the feasibility of such a replacement as well as the availability of more ancient specimens is required to conclude whether the African introgression hypothesis is a viable one and to refine its time boundaries.

Here we use analytical approaches to explore the possibility of the proposed mtDNA replacement and we introduce a novel Neanderthal mtDNA lineage from a femur found at the Hohlenstein–Stadel (HST) cave of the Swabian Jura in southwestern Germany^15^ (Fig. 1a,b). We use HST to further explore the mtDNA genetic diversity among archaic humans and describe their phylogenetic relationships with modern humans, to infer Neanderthal demographic processes across the Middle and Late Pleistocene.

## Results

### Archaeology and stable isotopes

**Figure 1:**
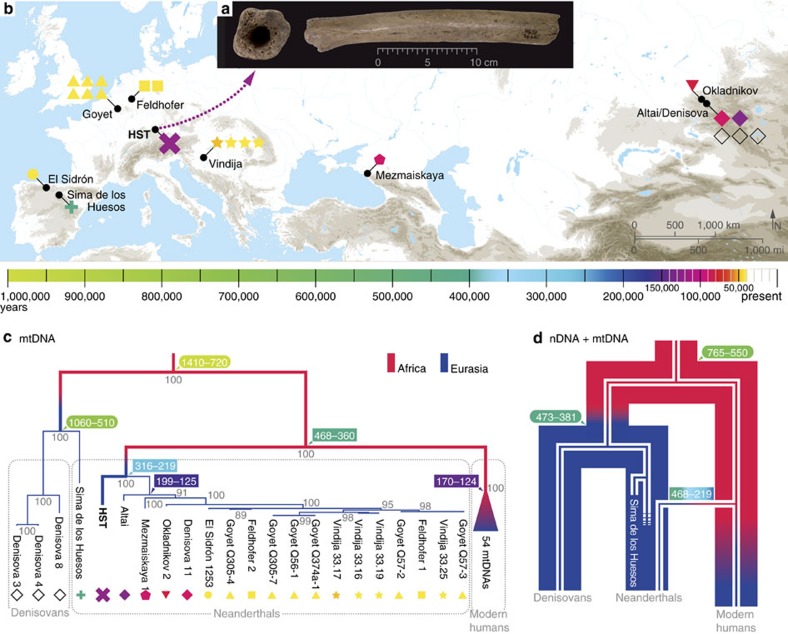
Archaic and modern humans' mtDNA and nDNA evolutionary scenarios. (**a**) Pictures of the HST femur, (**b**) map of archaeological sites where complete mtDNA from archaic humans were reconstructed, (**c**) maximum parsimony tree of 54 modern human (collapsed), 18 Neanderthal, 3 Denisovan and 1 Sima de los Huesos mtDNAs built with coding region only and 98% partial deletion. Grey node numbers refer to bootstrap support after 1,000 iterations. Tree rooted with a chimpanzee mtDNA (not shown). (**d**) Schematic comparison of the nDNA (wide lines) with the mtDNA (thin lines) phylogenies of Neanderthals, Denisovans and modern humans. In **c**,**d**, colour legend for individual symbols and node numbers is illustrated in the horizontal time line. Node numbers in rectangular boxes are divergence times estimated in this study (Table 1), while in oval boxes are dates estimated in Prüfer *et al*.^5^ and Meyer *et al*.^3^ in thousand years before present. Red and blue tree branches represent supposed African and Eurasian distribution, respectively.

The HST specimen is a right femur shaft circa 25 cm long displaying archaic hominin morphology, affected by heavy mineralization and gnawing by a large carnivore on both sides^15^ (Fig. 1a). During excavations in 1937, it was found in a black clayey layer with Middle Paleolithic artefacts known as the Black Mousterian based on the sediment colour and the cultural assignment of the technocomplex retrieved in the stratigraphic unit, which is associated throughout Europe with Neanderthals^16^. The femur is the sole archaic human fossil originating from a Mousterian context, not only at the site but in the entire Swabian Jura region^17^ (Supplementary Note 1).

Direct radiocarbon dating attempts have resulted in inconsistent results (Supplementary Note 2), suggesting that the bone may be suffering from modern ^14^C contamination and is possibly beyond the detection limit of this dating method. Isotopic analyses performed on the collagen of the femur revealed considerably lower δ^13^C and δ^15^N values than those reported for late Neanderthals from western and central Europe (Supplementary Table 1 and Supplementary Fig. 1)^18^. Moreover, collagen from two faunal remains recovered from the same stratigraphic unit of HST was analysed. ZooMS analyses^19^ confirmed the morphological assignment to red deer and radiocarbon dating resulted in an age range beyond this dating method (Supplementary Table 1 and Supplementary Note 2). Both deer specimens provided notably lower δ^13^C values compared to cervids from open steppic environment^20^ (Supplementary Fig. 2). The ecological background of the HST femur and deer specimens are, therefore, equivalent and indicate a more forested and closed environment compared to the habitat of late Neanderthals in northwestern Europe^18^,^21^.

### Ancient DNA retrieval and consensus reconstruction

The femur shaft was sampled from the proximal diaphysis longitudinally to the cortical bone, at the opposite site of the previous sampling for radiocarbon dating. DNA was extracted from 130 mg of bone powder^22^, immortalized in a double-stranded library^23^ and hybridized to modern human mtDNA probes^24^. The enriched library was sequenced and between 12,750 and 12,848 DNA reads were successfully aligned to four reference sequences with the same mapping parameters (see Methods section): the reconstructed MRCAs of Neanderthal and *Homo sapiens* mtDNA (reconstructed Neanderthal reference sequence (RNRS) and reconstructed Sapiens reference sequence (RSRS))^25^, the Neanderthal-type specimen mtDNA (Feldhofer1)^26^ and the present-day human mtDNA reference (revised Cambridge reference sequence (rCRS))^27^, respectively. A consensus sequence for each of the four references was reconstructed with endoCaller implemented in the software schmutzi^28^, followed by visual inspection to confirm the called polymorphisms (see Methods section). Using the RNRS as reference sequence resulted in the highest number of mapped reads and ∼35-fold average mtDNA coverage. Around 50% of mtDNA fragments were damaged at the molecule termini with an average length of ∼43 bp, both displaying the degradation patterns typical for ancient DNA (aDNA)^29^ (Supplementary Table 2 and Supplementary Fig. 3). When comparing the four consensus sequences obtained by mapping against the different references we observed the influence of reference biases in reconstructing the HST mtDNA (Supplementary Fig. 4a). After manual inspection of the inconsistent positions, we identified RNRS as the reference producing a consensus sequence closest to the endogenous mtDNA (see Methods section). However, mapping bias disappeared when excluding from the alignment the highly variable D-loop region (Supplementary Note 3 and Supplementary Fig. 4b). Following a more conservative approach subsequent Bayesian and phylogenetic analyses were performed using the reconstructed HST mtDNA coding region. The phylogenetic comparison with 54 modern humans, three Denisovan and an extended dataset of 17 Neanderthal mtDNA sequences revealed a closer relationship of the femur’s mtDNA to Neanderthals. However, the HST mtDNA revealed a short phylogenetic branch length and fell basal to all other Neanderthal individuals, representing the deepest diverging lineage among Neanderthal mtDNAs discovered to date (Fig. 1c, Supplementary Fig. 5 and Supplementary Fig. 6).

The same HST genetic library before mtDNA capture was also sequenced through a shotgun approach. Only 0.46% of the over half million reads were aligned to the human reference genome (hg19) despite choosing a highly sensitive mapping parameters to account for aDNA damage and divergence from the reference sequence (Supplementary Table 3 and Methods section).

### Contamination estimates

Three measurements were performed to estimate the level of present-day human contamination in the isolated mtDNA reads. The first approach is based on the assumption that aDNA is damaged, whereas contaminant DNA is less affected by this chemical modification (contDeam^28^). One molecule end is conditioned to exhibit damage while deamination levels are measured at the opposite end of the fragment. The discrepancy between the unfiltered and conditioned damage levels implied a contamination of 9.5–11.5% from modern human fragments (Supplementary Table 2).

This estimate is used as prior in an iterative likelihood approach in which mtDNA reads are compared to a data set of 256 Eurasian modern mtDNA sequences to refine the level of contamination (mtCont^28^). According to this second method, 9–11% of the bases aligned to the rCRS turned out to be of contaminant origin.

Third, we identified mtDNA diagnostic positions, as the nucleotides where the reconstructed HST complete sequence differed from more than 99% of 311 worldwide mtDNAs^30^. From 123 differences, only 11 transversions were considered to avoid the risk of wrongly classifying damage that is typically seen as transitions^31^, as real substitutions. For each transversion we counted the total number of fragments harbouring a base consistent with the HST consensus over the ones showing the almost fixed modern human variant. The mtDNA contamination was estimated to be 5.4–12.2%.

Overall, the three approaches consistently returned modern human DNA contamination levels with an upper value of ∼12%. This may be associated with the presence of modern collagen contamination that possibly resulted in inconsistent radiocarbon dates. While mtDNA consensus sequences can be confidently reconstructed with such contamination proportions^32^, nuclear DNA analyses would be highly affected.

### MtDNA Neanderthal diversity

**Figure 2:**
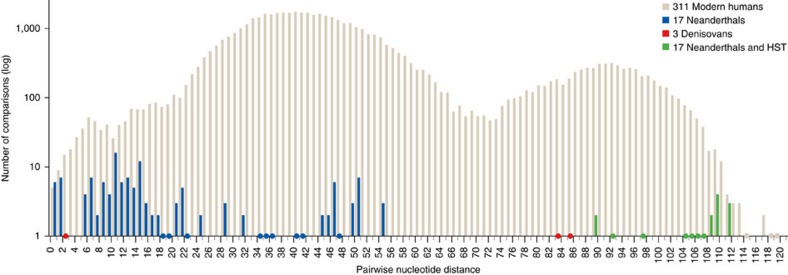
Archaic and modern humans' mtDNA diversity. The pairwise nucleotide distance over its frequency (in logarithmic scale) is measured among 311 worldwide modern human, 17 Neanderthal, 3 Denisovan and 18 Neanderthal (including HST) mtDNAs. Points on the *x* axis represent one sequence pair comparison.

In a previous study^2^, the mtDNA diversity among seven Neanderthals, three Denisovans and 311 modern humans were compared through the Watterson’s estimator *θ*_w_, resulting in the lowest mtDNA distance within Neanderthals. The value decreased even further when 10 additional Neanderthal mtDNAs available in the literature were included (1.37 × 10^−3^), which confirms the small population size of late Neanderthals^26^ (Supplementary Table 4). However, by adding the HST mtDNA in the Neanderthal group the *θ*_w_ estimation almost doubled to 2.50 × 10^−3^. Although the value is still below the results obtained from the three Denisovan sequences (3.46 × 10^−3^), the HST mtDNA exhibits an average pairwise nucleotide distance to the other Neanderthal mtDNAs of 104 (89–111) positions (Fig. 2 and Supplementary Table 5). These values are greater than among any Denisovan mtDNA pair and are in the upper range of the modern human worldwide pairwise distance distribution (Fig. 2). This shows that HST belongs to a mtDNA branch highly divergent from the one represented in other Neanderthals (Altai branch) and overall Neanderthal mtDNA diversity was larger than that assumed previously.

The Neanderthal mtDNA effective population size (*N*_e_) through time was estimated in a Bayesian statistical framework^33^ under the simplified assumption they belonged to a panmictic population with a fixed mutation rate previously calculated with ancient modern human mtDNAs as calibration points^10^ (Supplementary Note 4). The reconstructed skyline plot describes a *N*_e_ reduction through Middle and Late Pleistocene, reaching the lowest mean value at around 42 ka (Supplementary Fig. 7). Subsequently, a steep population expansion appears to have occurred before the Neanderthal extinction, in accordance with the reported analyses of chromosome 21 of the Vindija late Neanderthal^14^.

### Molecular dating analyses

To estimate the molecular age of HST and other undated Neanderthal mtDNAs as well as the temporal range of MRCAs (TMRCAs) on the mtDNA tree, we performed a Bayesian dating analysis as implemented in BEAST v.1.8.1 (ref. 33). A multiple genome alignment of the coding region from 54 modern humans, 18 Neanderthals and 1 Denisovan mtDNA were tested for a strict and uncorrelated lognormal relaxed clock under both a constant size and a Bayesian skyline tree prior (see Methods section). As reported above, a fixed mutation rate was selected for the coding region^10^ with the addition of eight dated Neanderthal mtDNAs as time anchors on the Neanderthal branch (Supplementary Table 6). The four model combinations were compared by stepping-stone and path sampling (PS) methods^34^. This analysis indicated that a skyline model associated with a strict rate variation among branches is the model that most adequately fits the data (Supplementary Table 7). In Table 1 we report the TMRCAs between Neanderthal and modern human mtDNAs and among modern human mtDNAs itself, which largely overlap with previously published studies^10^,^11^. We further estimate the divergence time between HST and all other Neanderthals to ∼270 ka (95% HPD 316–219 ka), while the TMRCA for the Altai branch was inferred to be ∼160 ka (95% HPD 199–125 ka).

Based on phylogenetic branch shortening, we then molecularly dated 10 Neanderthal sequences that had not been radiocarbon dated previously or were considered beyond the radiocarbon dating detection limit (Table 1). The two oldest mtDNAs were HST with an age of 124 ka (95% HPD 183–62 ka) and Altai Neanderthal with an age of 130 ka (95% HPD 172–88 ka). Notably, the mean value for the latter individual largely overlaps with the inferred age of 136–129 ka from its high coverage nuclear genome analyses, when applying recent estimates of the human mutation rate^5^.

### Exploration of putative Neanderthal mtDNA replacements

The probability that the initial Denisovan-like Neanderthal mtDNA present in Eurasia was totally displaced by an incoming lineage^8^ is dependent not only on the admixture rate but also on the size of the introgressing population compared to the local one (Supplementary Fig. 8). When considering the effective population size history estimated with the Bayesian skyline method (Supplementary Fig. 7), the probability that all Neanderthal mtDNA originated from an introgression event is almost directly proportional to the admixture rate (Supplementary Fig. 9 and Supplementary Note 5). Moreover, assuming that a complete mtDNA replacement took place, we estimated under neutrality^35^ (see Methods section) the mean time period necessary for such a lineage to reach fixation given a mtDNA introgressing fraction below 20% and initial effective population size (*N*_e_) up to 10,000 units (Supplementary Table 8). We molecularly dated the split of the HST lineage from other Neanderthal mtDNAs to ∼270 ka (Table 1) that represents the minimum time available for the Late Pleistocene branch to replace the pre-existing Denisovan-like mtDNA. From our calculations, if *N*_e_ was <5,000 units, a mean temporal interval of 300 ka is sufficient for an incoming mtDNA lineage below 0.1% in frequency to drift up to fixation.

Within the Late Pleistocene mtDNA clade, we explored if the HST mtDNA branch might have survived long after the estimated molecular age of the HST femur. All complete Neanderthal mtDNAs were combined with sequences from published hypervariable regions (HVRI) of four additional Neanderthal individuals. We identified the Valdegoba sequence (JQ670672) sharing three derived mutations with HST and falling on the same branch in a HVRI tree (Supplementary Fig. 10 and Methods section). This specimen was found on the Iberian Peninsula and dates to 48,400±3,300 ^14^C years BP^36^. Although a complete mtDNA would be necessary to measure the total mtDNA distance between HST and Valdegoba, this finding might suggest that the HST branch was found during the Late Pleistocene as far as western Europe. Based on the geographical and temporal distributions of HVRI sequences, it was proposed that the Neanderthal population in western Europe underwent a demographic turnover followed by a subsequent recolonization^36^. Under that scenario, the HST lineage would have been largely replaced towards the end of the Neanderthal temporal range by mtDNAs descendants on the Altai branch.

## Discussion

The African introgression hypothesis suggests that Late Pleistocene Neanderthal mtDNAs originated through gene flow from an African source^8^, which we constrain taking place more than ∼270 ka (Table 1). Our analytical calculations (Supplementary Table 8 and Supplementary Note 5) show that this event is plausible even if the introgressing lineage represented a minimal proportion of the initial gene pool. This scenario reconciles the discrepancy in the nDNA and mtDNA phylogenies of archaic hominins and the inconsistency of the modern human–Neanderthal population split time estimated from nDNA and mtDNA (Fig. 1d). Under this demographical model, the Denisovan mtDNA type was common among early Neanderthals in Eurasia (for example, Sima de los Huesos) and was then largely replaced by an introgressing African mtDNA that evolved into the Late Pleistocene Neanderthal mtDNA type. While the upper bound for the time of this putative gene flow event would be the divergence time between Neanderthal and modern human mtDNAs, here dated to 413 ka (95% HPD 468–360 ka), the lower temporal limit was represented so far by the ∼160 ka TMRCA of all published Neanderthal mtDNAs (Table 1). However, the finding of the deeply diverged HST lineage splitting from the Altai branch, ∼270 ka, sets an older lower boundary for the time of this admixture event. An alternative but less parsimonious scenario is that both HST and Altai mtDNA lineages reached Eurasia independently after diverging inside Africa. In that case the suggested introgression event might have occurred later but most likely before 160 ka, our estimated date for the start of the Altai branch diversification (Fig. 1c and Table 1).

The presence of modern human admixture into archaic humans has already been detected in the high coverage Neanderthal genome from the Altai region but not in sequences of chromosome 21 of two Neanderthals from Spain and Croatia^14^. The authors therefore suggested that a genomic contribution estimated between 0.1 and 2.1% occurred after the divergence of Altai from other late Neanderthals. However, there is a high level of uncertainty around the time of the inferred gene flow event since only one high coverage Neanderthal nuclear genome has been analysed so far. Moreover, the divergence time of the introgressing African population was estimated to date before or right after the TMRCA of modern-day humans (∼200 ka)^14^, while the mtDNA coalescence time between Neanderthals and modern humans is calculated at least twice as old (∼400 ka). The evolutionary scenario responsible for providing the mtDNA to the Late Pleistocene Neanderthals might have been an even earlier Middle Pleistocene gene flow from Africa, occurring in a time interval that we date between 413 and 268 ka (460–219 ka including upper and lower 95% HPD). It should be highlighted that this additional genomic contribution might have already been accounted for in ref. 14, which effectively measures the total amount of African introgression into Neanderthals after their split from Denisovans (473–381 ka; ref. 5).

The phylogenetic branch length of mtDNA sequences from 10 non-dated Neanderthal individuals was considered in BEAST, to assess individual molecular ages spanning from 130 to 40 ka. Although it is not known if the mtDNA mutation rate in modern humans is comparable to that of Neanderthals (Supplementary Note 4), molecular dating can at least be used to provide relative ages when the radiocarbon absolute chronometric method is not applicable. After the Altai mtDNA, HST is estimated to be the second oldest mtDNA with an age of 124 ka (95% HPD 183–62 ka). This wide temporal interval largely overlaps with the Marine Isotope Stage 5 (MIS 5: ∼130–73 ka)^37^. After its initial interglacial period (MIS 5e), central Europe was characterized by climatic fluctuations resulting in forestation cycles (MIS 5c/5a) alternated with the development of steppe-tundra biomass (MIS 5d/b)^38^. The stable isotopic δ^13^C and δ^15^N values of the archaic femur collagen and associated faunal remains support a more temperate, forested rather than a colder, steppe environment and is therefore consistent with an ecological context during the early warm phases of the last glaciation^17^.

Despite having only a single complete mtDNA on the HST lineage, the two highly differentiated Neanderthal mtDNA branches suggest higher mtDNA diversity during the Middle Pleistocene, which then declined during the Late Pleistocene (Supplementary Table 4). This observation is also supported by the steady decline in mtDNA effective population size displayed in the skyline plot before a steep growth in late Neanderthal population sizes (Supplementary Fig. 7). Studies focusing on the demographic patterns of late Neanderthals who overlapped with the earliest modern humans in Europe are of key importance to understand population dynamics and interactions between archaic and modern humans.

In conclusion, the HST mtDNA provided insights into the mtDNA diversity of Neanderthal populations through the Middle and Late Pleistocene. Its deep divergence time allowed us to further constrain the lower boundary for the time of the proposed African mtDNA gene flow into Neanderthal populations. The temporal corridor for this introgression event between 460 ka and 219 ka is compatible with the evidence of archaeological similarities between Africa and western Eurasia during the Lower to Middle Paleolithic transition^39^ and potentially may explain the dissimilarities in Middle Paleolithic industries between eastern and western Eurasia. Environmental changes across this time span might have facilitated a hominin expansion out of Africa and potentially spread cultural innovations such as the Levallois technology into Eurasia^40^. Alternatively, other scenarios such as multiple inventions of similar technologies by various hominin groups may explain the complex tapestry of technological variability during the late Middle Pleistocene.

Nuclear data from the HST femur would be pivotal in assessing its genomic relationships with Neanderthals, Denisovans and modern humans. However, the scarce preservation of HST endogenous DNA in combination with high level of modern human contamination challenge the retrieval of its complete genome. Analyses of high-quality nDNA from more than one well-preserved Neanderthal individual are necessary to detect the consequences of African admixture into archaic human populations.

## Methods

### aDNA lab work

aDNA work was performed in the dedicated facilities of the Institute for Archaeological Sciences in Tübingen, Germany. The HST femur was first irradiated with ultraviolet light on the selected sampling area and then drilled with a dentist drill along the cortical bone. A total of 130 mg of bone powder went into the DNA extraction following an established protocol^22^. DNA was eluted in 100 μl of TET and 20% of the extract (GX35) was used to build a double-stranded genetic library (GA87)^23^. The total copies in the resulting library were measured with quantitative PCR (qPCR) (7.53 × 10^9^ copies). They were split into four 100 μl indexing PCR reactions with 10 cycles where an individual index pair (8 bp each) was assigned to create a unique double-indexed library^41^. The total copies were measured again via qPCR (2.60 × 10^11^ copies) and the reaction efficiency was calculated by dividing the number of total molecules after indexing by the number of total molecules before indexing PCR. An aliquot of two-fifth of the indexed library was split into two reactions that were amplified for seven cycles each with AccuPrime Pfx DNA polymerase. The PCR products were purified over a single MinElute spin column and the concentration after amplification was quantified to 286 ng μl^−1^ on an Agilent 2,100 Bioanalyser DNA 1,000 chip. Extraction and library negative controls were carried along the workflow and treated equally.

The amplified library was enriched for mtDNA using modern human baits as reported by Maricic *et al*.^24^. This protocol has been previously used to successfully capture complete Neanderthal mtDNA genomes^4^. Four hundred nanograms of the amplified library were pooled with the same amount of four other libraries for a total of 2,000 ng and captured with 500 ng of mtDNA probes. After purification, the isolated molecules were quantified with qPCR (4.84 × 10^6^ copies) and reamplified for 20 additional cycles as described above. The captured pool as well as the uncaptured GA87 library was quantified with Agilent 2,100 Bioanalyser DNA 1,000 chip, diluted to 10 nM and sequenced with other equimolar libraries on an Illumina HiSeq2500 Rapid run via 2 × 100+8+8 cycles and on an Illumina NextSeq500 run via 2 × 75+8+8 cycles.

### Sequence processing and mtDNA consensus reconstruction

Sequenced molecules were converted from bcl to fastq files and reads containing the defining library indexes were binned in an individual folder. The EAGER pipeline was used for all subsequent data processing^42^. Initially adapter and index sequences were trimmed off. Only merged reads where forward and reverse reads overlapped by at least 10 bp were retained. Shotgun sequences above 30 bp were aligned to the complete human genome (hg19) with Burrow–Wheeler Aligner (parameters −*n* 0.01 and seeding off) to calculate the percentage of human DNA. Duplicates and reads with mapping quality below 30 were discarded to estimate damage patterns and average fragment length (Supplementary Table 3). From the total of ∼3 Ma paired-end reads sequenced after mtDNA capture, 89.31% were successfully merged and fragments below 30 bp length were further discarded for mapping. The resultant ∼1.3 Ma merged reads were aligned to four reference mtDNA sequences: the RSRS^25^, the rCRS^27^, the Neanderthal Feldhofer 1 sequence^26^ and the RNRS originally proposed in Behar *et al*.^25^ and later updated when the more basal Altai mtDNA was published^5^. The rCRS and Feldhofer 1 references are two derived mtDNA sequences on the modern human and Neanderthal branch, respectively. Instead, RSRS and RNRS represent the MRCA mtDNA for modern humans and Neanderthals, respectively. Reads were mapped using Burrow–Wheeler Aligner^43^ with identical parameters (−*n* 5 and seeding off) for all four references, in combination with a tool able to consider the circularity of mtDNA as part of EAGER. The percentage of target DNA was calculated by dividing the total number of input reads by the reads mapping to each mtDNA reference. Duplicates with the same start and end coordinates were removed and the duplication factor was measured by dividing the total number of reads mapping before by the total number of reads mapping after duplicate removal. All fragments with map quality below 30 were removed to estimate the average mtDNA coverage. The resulting molecules were also used to calculate average fragment length and deamination patterns^44^ (Supplementary Fig. 3). Statistics for each processing step of the four reference sequences are reported in Supplementary Table 2.

Consensus reconstruction was performed in a two-step approach. First, schmutzi^28^ was used to infer the endogenous sequence. An internal program of the software package (contDeam) was first run to calculate the endogenous deamination rate and a contamination prior. To each nucleotide a base likelihood value was assigned incorporating damage, base quality and mapping quality information in a Bayesian framework^28^. The endogenous consensus was then determined by endoCaller after the first iteration of the program. No cutoff to the nucleotide posterior probability was selected resulting in base called even in positions covered with only one fragment. This produced a consensus sequence with three unassigned positions.

Second, the four consensus sequences, one from each reference, were visually compared in Geneious 8.1.7 (http://www.geneious.com)^45^. A multiple genome alignment was produced and each of the 19 inconsistent positions between the four consensus was evaluated. We imported the bam files in Geneious and for each read covering those positions we inspected if they also overlapped neighbouring confidently assigned SNPs (for example, called in all four consensus). Fragments containing such SNPs were considered as endogenous, whereas reads containing the alternative allele were considered as contaminants. In every case, the consensus sequence reconstructed after mapping against the RNRS reference was found to exhibit the endogenous base. This confirms that mapping against a reference sequence that is phylogenetically closer to the consensus sequence increases mapping accuracy (see Supplementary Note 3). Using the same criterion described above we then manually screened the RNRS mapped consensus and edited the following positions according to rCRS coordinates. Two miss-mapped insertions were removed (pos. 247delT and 16184delA), two uncertain positions with low coverage were edited (A189G and A4296N) and two regions covered with only contaminant reads were masked (pos. 203–214Ns and 5486–5508Ns). We additionally masked the known troublesome regions of poly-C (pos. 303–315) and poly-AC (518–524) stretches^46^. Combining the two approaches resulted in a total of 59 unassigned positions in the final consensus sequence that was used for phylogenetic (Supplementary Fig. 5) and mtDNA diversity analyses. We then generated a more conservative consensus by setting a coverage cutoff to twofold. The resulting mtDNA sequence exhibits 81Ns, but none of the 22 additional unassigned bases overlapped with polymorphic positions within the known Neanderthal mtDNA diversity. Therefore, the tree topology and mtDNA diversity within Neanderthals was not affected.

### Contamination with modern human mtDNA

We followed three different approaches to estimate modern human contamination levels in the isolated mtDNA. The first method is implemented in contDeam^28^ and it relies on deamination patterns. This program works on two assumptions: that modern human DNA contamination presents no damage and that the damage at one end of a molecule is independent of the one at the other end. Reads with deamination at the 5′ end are selected and the deamination rate is measured at the 3′ end and vice versa. The calculated value is supposed to represent the true damage signal of the endogenous mtDNA fragments. A contamination estimate is then computed as the percentage of undamaged reads necessary to shift the damage rate from the endogenous value to the one initially calculated on all fragments. We obtained an estimate ranging from 9.5% to 11.5% (for all four references combined) that could be an underestimation of the real contamination level if the contaminant DNA is also damaged. However, simulations have shown that this effect is marginal if the deamination rate of the endogenous DNA is over 50% at the molecule termini^28^, like observed for HST mtDNA fragments (Supplementary Fig. 3).

The second method makes use of the probabilistic iterative method implemented in schmutzi^28^. The program was run with the fallowing parameters: ‘-- notusepredC -uselength’. The contamination estimate is performed with the tool mtCont using sites where the endogenous sequence differs from a non-redundant data set of 256 Eurasian mtDNAs. For this method, we used the mtDNA reads mapping to the rCRS reference according to which base frequencies of the comparative data set are calculated. While contDeam measured contamination rate on a fragment level, mtCont provided an estimate at a base level of 9–11%.

The third method is based on the diagnostic positions where the reconstructed HST consensus differs from present-day worldwide mtDNAs. All polymorphic positions with a frequency above 1% in a data set with 311 worldwide mtDNAs are not considered. We then identified 123 positions where HST has a different base compared to more than 99% of the 311 mtDNAs. Of those, we restricted the analysis to only transversions (positions 2,831, 6,265, 7,105, 9,328, 9,354, 11,457, 13,761, 13,878, 14,457, 14,925, 16,138). Of the total 262 reads covering the 11 positions, 239 reads presented the endogenous base, while 23 reads the contaminant variant. This resulted in a contamination rate of 8.8% (confidence interval 95%, 5.4–12.2%). The last approach^30^ provides a direct measure for the proportion of contaminant fragments and overlaps with the two previous estimates.

### Phylogenetic analyses

To further explore the maternal relationships of the HST mtDNA with other archaic and modern human mtDNAs we compared the phylogenetic placement of the HST consensus sequence with and without D-loop with 17 Neanderthal, 54 modern human^47^, three Denisovan^1^,^2^ and Sima de los Huesos^3^ mtDNAs, plus a chimpanzee mtDNA (GenBank: X93335.1) to root the tree. Two maximum parsimony trees with the 77 mtDNAs and 1,000 iterations each were built, one including the whole molecule and 97% partial deletion (16,536 positions) and one with the coding region only and 98% partial deletion (15,417 positions) (Fig. 1c and Supplementary Fig. 5). The topology of both trees is consistent with HST diverging from the Neanderthal branch more basally than any other sequence and presenting a short phylogenetic branch length.

We also tested in Modelgenerator^48^ the same multiple genome alignment with only the coding region, including missing sites but not gaps. The substitution model that best fits the data (AIC1) was GTR with invariant sites and γ-distributed correction for rate heterogeneity. These parameters were selected in MrBayes^49^, used to build a Bayesian phylogenetic tree (Supplementary Fig. 6). Fifty millions iterations of the Markov chain Monte Carlo (MCMC) were run with 10,000 sampling interval. From the total trees, the first 10% were removed as burn-in and a summarized tree was generated. All major branches show posterior support of 1 and confirmed the maximum parsimony trees topology.

We finally explored the diversity of the Neanderthal HVRI including four additional sequences for which only HVRI was available in GenBank (Valdegoba JQ670672, Scladina DQ464008, Teshik-Tash EU078679, Monti Lessini DQ836132). We aligned them to the HVRI of 17 complete Neanderthal mtDNAs (excluding Denisova 11 because of several unassigned positions in the HVRI), three Denisovan mtDNAs and the rCRS using MUSCLE^50^. We then built a maximum parsimony phylogeny in MEGA6 (ref. 51) with complete deletion (105 positions) and 1,000 bootstrap iterations (Supplementary Fig. 10).

### Mitochondrial DNA diversity

The pairwise nucleotide distance among Neanderthals with (*n*=18) and without HST (*n*=17), Denisovan (*n*=3) and modern human (*n*=311) mtDNAs was calculated in MEGA6. For this analysis, we used the complete mtDNAs sequences and the number of differences between them was counted with pairwise deletion where all unassigned positions were removed for each sequence pair. We plotted the pairwise nucleotide distance against their frequencies for each of the four data sets (two Neanderthals, Denisovan and modern human) in Fig. 2. We also reported the average distance (and minimal–maximal values) of 311 modern humans, three Denisovans, Sima de los Huesos and 17 Neanderthals to the HST complete mtDNA (Supplementary Table 4). The lowest distance is with Neanderthals, followed by modern humans, Sima de los Huesos and Denisovans in agreement with the phylogenetic assignment. However, the nucleotide distances between HST and other Neanderthals are the largest observed among Neanderthals (89–111 nucleotides). These values are higher than between Denisova 3–Denisova 4 and Denisova 8 and around the uppermost edge among 311 worldwide mtDNAs (Fig. 2).

We further measured the mtDNA diversities of the enlarged Neanderthal mtDNA data set with the Watterson’s estimator, as reported in ref. 2. We first prepared a multiple genome alignment of Neanderthal mtDNAs both including HST (18 sequences) and excluding HST (17 sequences) using MUSCLE. Then, the number of segregating sites (*K*) was estimated with DNA Sequence Polymorphism (DnaSP) v.5.10.01 (ref. 52). Finally, *θ*_w_ was calculated as follows: *K*/*a*_n_/16,595, where *a*_n_ is Σ_*i*=1_^*n*−1^1/*i* to take in consideration the number of mtDNA sequences in each data set. Adding HST to the 17 Neanderthal mtDNAs, the number of segregating sites almost doubled (from 78 to 145), whereas *θ*_w_ increased from 1.37 × 10^−3^ to 2.50 × 10^−3^ (Supplementary Table 4). The latter value is closer to the mtDNA diversity estimated within three Denisovan mtDNAs (3.46 × 10^−3^)^2^.

### BEAST analyses

We used the software package BEAST v.1.8.1 (ref. 33) to both estimate the divergence times between and within modern and archaic humans as well as to track the changes in the maternal effective population size (*N*_e_) of Neanderthal mtDNAs through time.

For the skyline analyses, we first created a multiple genome alignment with only the mtDNA coding region of 18 Neanderthal mtDNAs and the rCRS as outgroup. We then removed from the alignment all columns where at least one mtDNA presented a gap or missing data, resulting in 15,345 positions. We run Modelgenerator v.85 (ref. 48) on our data set to identify Tamura-Nei 93 with a fixed fraction of invariable sites as the best-supported model. We set a fixed mutation rate (1.57 × 10^−8^ μ site^−1^ year^−1^)^10^ calculated for the coding region of modern humans with ancient mtDNAs as calibration points (Supplementary Note 4). As tree prior we selected the Bayesian skyline coalescent with 10 as group number and piecewise linear as the skyline model. We tested both a strict clock and an uncorrelated lognormal-distributed relaxed clock. For both models three MCMC runs with 50,000,000 iterations were run, with 10,000 sampling frequency. We discarded 10% of the states from each run as chain burn-in and then combined the three independent runs for both models using LogCombiner v.1.8.1 (included in the BEAST package), resulting in a total of 135 millions iterations. The two models were compared with a marginal likelihood estimation using path sampling (PS) and stepping-stone sampling (SS)^34^. The skyline tree prior in combination with a strict variation among tree branches performed better according to PS, while lognormal-distributed relaxed clock was best supported according to SS (Supplementary Table 7). The strict clock provided higher effective sample size (ESS) values because of earlier chain convergence; therefore, it was the chosen model to reconstruct a skyline plot for the 18 Neanderthal mtDNAs. We used Tracer v.1.6 selecting linear change as Bayesian skyline variant and a default of 100 as number of bins. In Supplementary Fig. 7 we report the mean *N*_e_ (black line) and the 95% HPD interval (purple lines) of the Neanderthal mtDNAs in logarithmic value on the *y* axis and the temporal range from 350 to 32 ka on the *x* axis. We observe a *N*_e_ reduction until around 42 ka followed by a rapid and short growth inversion, predating the Neanderthal disappearance.

For the dating analyses, we instead used a data set composed of 18 Neanderthal, 54 modern humans and 1 Denisovan mtDNAs as outgroup. As described above, we removed the D-loop from the alignment and further excluded all positions containing gaps and missing data for a total of 15,334 positions. The best-supported model for this data set was again Tamura–Nei 93 with invariable sites as tested in Modelgenerator v.85. The same fixed mutation rate was selected and tip dates were indicated for the eight dated Neanderthal mtDNAs (Supplementary Table 6). The date for all 54 mtDNA was kept as zero, while a range between 30 and 500 ka (initial value 50 ka) was given for all the undated Neanderthals. We tested two models of rate variation within branches: a strict clock and an uncorrelated lognormal-distributed relaxed clock. We also investigated two different tree priors: a Bayesian Skyline coalescent and a constant population size. As before, for each of the four model combinations MCMC was run three times with 50,000,000 iterations, sampling frequency 10,000 and 10% burn-in. The resulting 135,000,000 iterations per model were combined using LogCombiner v1.8.1. Best-supported model assessment was again implemented with marginal likelihood estimation using PS and SS. The strict molecular clock with the skyline tree prior provided higher likelihoods than the three other tested models (Supplementary Table 7).

### Modelling the mitochondrial replacement

Under neutrality and assuming the Eurasian Neanderthal effective population size (*N*_e_) to be relatively small (that is, <10,000 *N*_e_ units), we calculated the mean time period necessary for an introgressing mtDNA lineage below 20% in frequency to reach fixation, when conditioning for that (Supplementary Table 8). This was computed using the following formula from Kimura and Ohta^53^, where *N* is the Eurasian Neanderthal *N*_e_ and *p* is the proportion of the introgressing mtDNA lineage: *T* (*p*)=−2*N* (1−*p*) ln(1−*p*)/*p* that was readapted from 4*N* for autosomes to 2*N* for mtDNA in ref. 35. Generations were converted into years assuming a generation time of 29 years (Supplementary Table 8). We also calculated the likelihood of a complete replacement as a function of the Neanderthal effective population size and the admixture rate from a branch basal to modern humans (Supplementary Note 5).

### Data availability

The HST mtDNA consensus sequence reported in this paper is available in GenBank with the accession code KY751400. All other data are available on request to the corresponding authors.

## Additional information

**How to cite this article:** Posth, C. *et al*. Deeply divergent archaic mitochondrial genome provides lower time boundary for African gene flow into Neanderthals. *Nat. Commun.* **8,** 16046 doi: 10.1038/ncomms16046 (2017).

**Publisher’s note:** Springer Nature remains neutral with regard to jurisdictional claims in published maps and institutional affiliations.

## Supplementary Material

Supplementary Information

## Figures and Tables

**Table 1:** Divergence times and molecular ages estimated in BEAST.

**Mitochondrial lineages**	**Mean value**	**95% HPD interval**
Modern humans—Neanderthals	412,930	467,720–360,230
HST—Altai branch Neanderthals	267,770	316,080–218,980
Altai—rest of Altai branch Neanderthals	160,480	198,800–125,410
San—rest of modern humans	146,730	169,520–123,650
Altai age	130,010	171,600–88,010
HST age	123,800	182,560–62,013
Mezmaiskaya 1 age	89,075	126,700–51,648
Denisova 11 age	88,244	113,760–63,840
Okladnikov 2 age	81,446	109,290–56,213
Vindija 33.17 age	48,809	57,157–40,532
Vindija 33.19 age	43,939	51,029–35,336
Vindija 33.25 age	42,996	52,305–34,450
Goyet Q374a-1 age	40,867	46,942–32,697
Goyet Q305-7 age	40,832	47,057–33,134

HST, Hohlenstein–Stadel.

Reported values derive from the skyline tree prior and strict molecular clock model that best fits the data (see Methods section).
