# Supplementary material for: Deeply divergent archaic mitochondrial genome provides lower time boundary for African gene flow into Neanderthals
Source: Nat Commun. 2017 Jul 4;8:16046. doi: 10.1038/ncomms16046 (PMC5500885; doi:10.1038/ncomms16046)
Supplement: Supplementary Information [file ncomms16046-s1.pdf]

Type of file: PDF
Size of file: 0 KB
Title of file for HTML: Supplementary Information
Description: Supplementary Figures, Supplementary Tables, Supplementary Notes and Supplementary References

Supplementary Figures

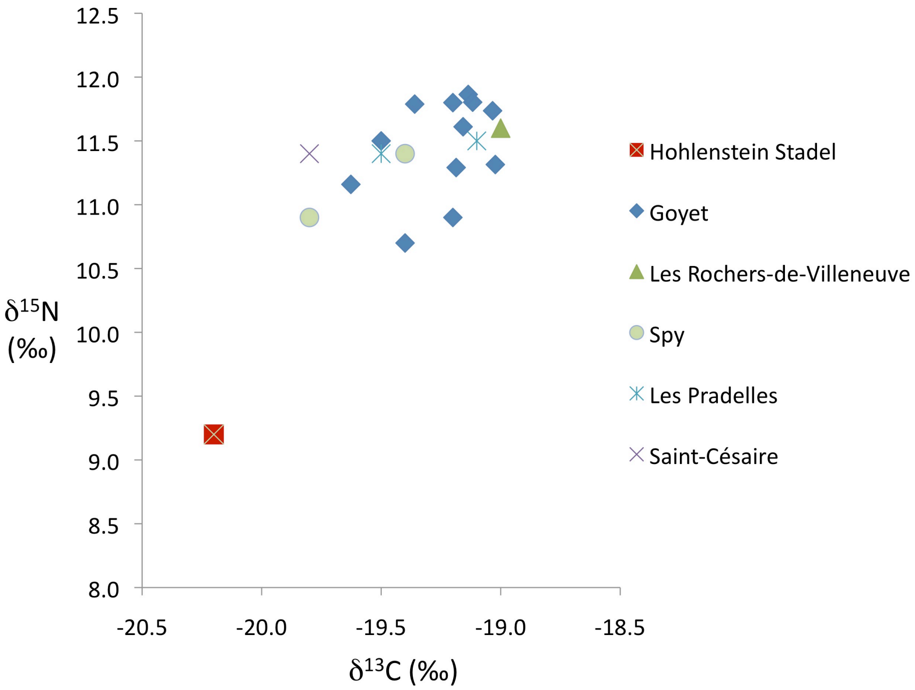

Supplementary Figure 1. Stable isotopic composition ($\delta^{13}C$ and $\delta^{15}N$ ‰) in collagen extracted from the HST femur (Supplementary Table 1) and other late Neanderthals[1-3].

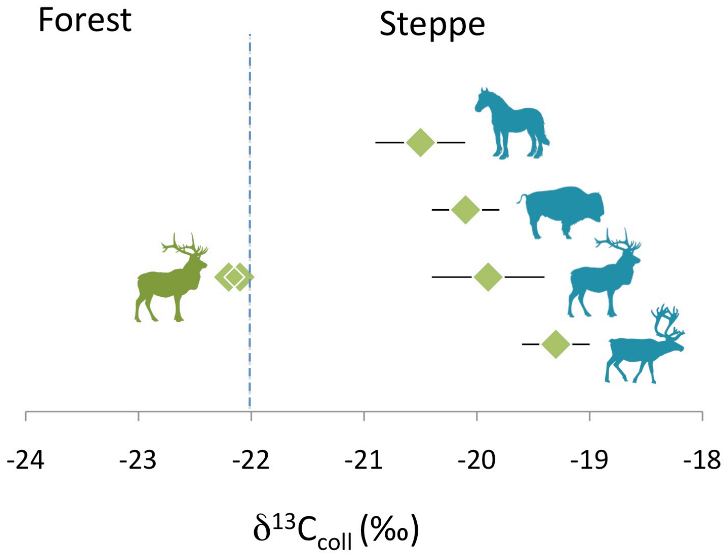

Supplementary Figure 2. Collagen $\delta^{13}C$ range of steppe herbivores (in blue from the top right: horse, bison, red deer and reindeer) compared to the $\delta^{13}C$ values of two red deer (in green on the left) from the same stratigraphic unit of HST suggesting for the latter a light forested environment (Supplementary Table 1). Error bars represent the standard deviation of the average $\delta^{13}C$ ‰ values.

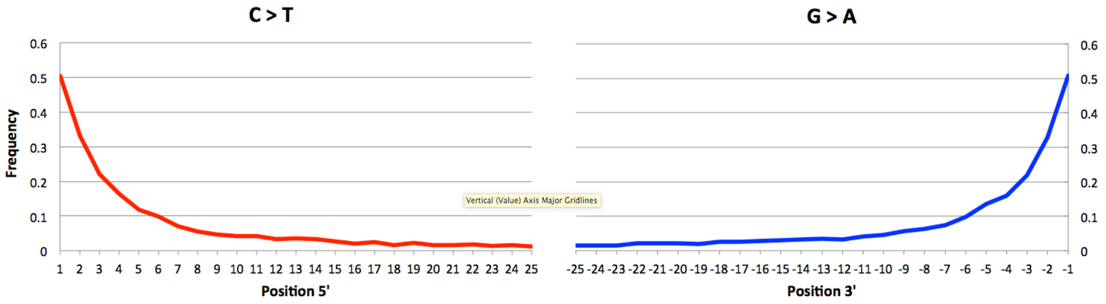

Supplementary Figure 3. Deamination patterns C to T at the 5' molecule end (in red) G to A at the 3' molecule end (in blue) in the mtDNA fragments mapped against the RNRS reference.

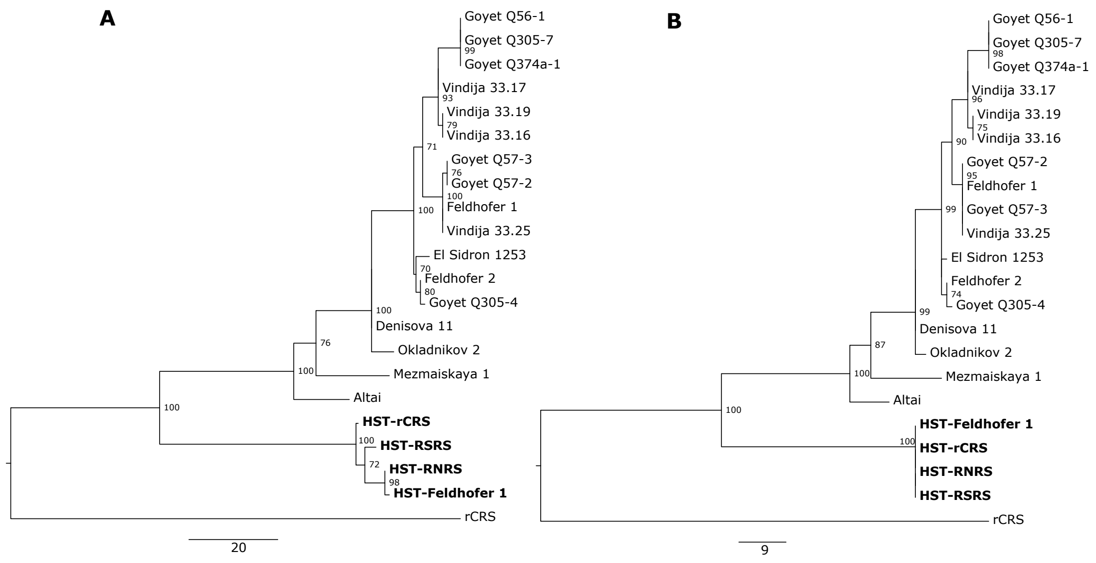

Supplementary Figure 4. Reference mapping bias. A) Maximum parsimony tree with complete mtDNA sequences using 1000 bootstrap iterations. HST consensus mtDNA show different branch lengths when mapping to four different references. B) Maximum parsimony tree with mtDNA coding region (without D-loop) and 1000 iterations as bootstrap support. Using only the HST coding region reduces reference mapping bias.

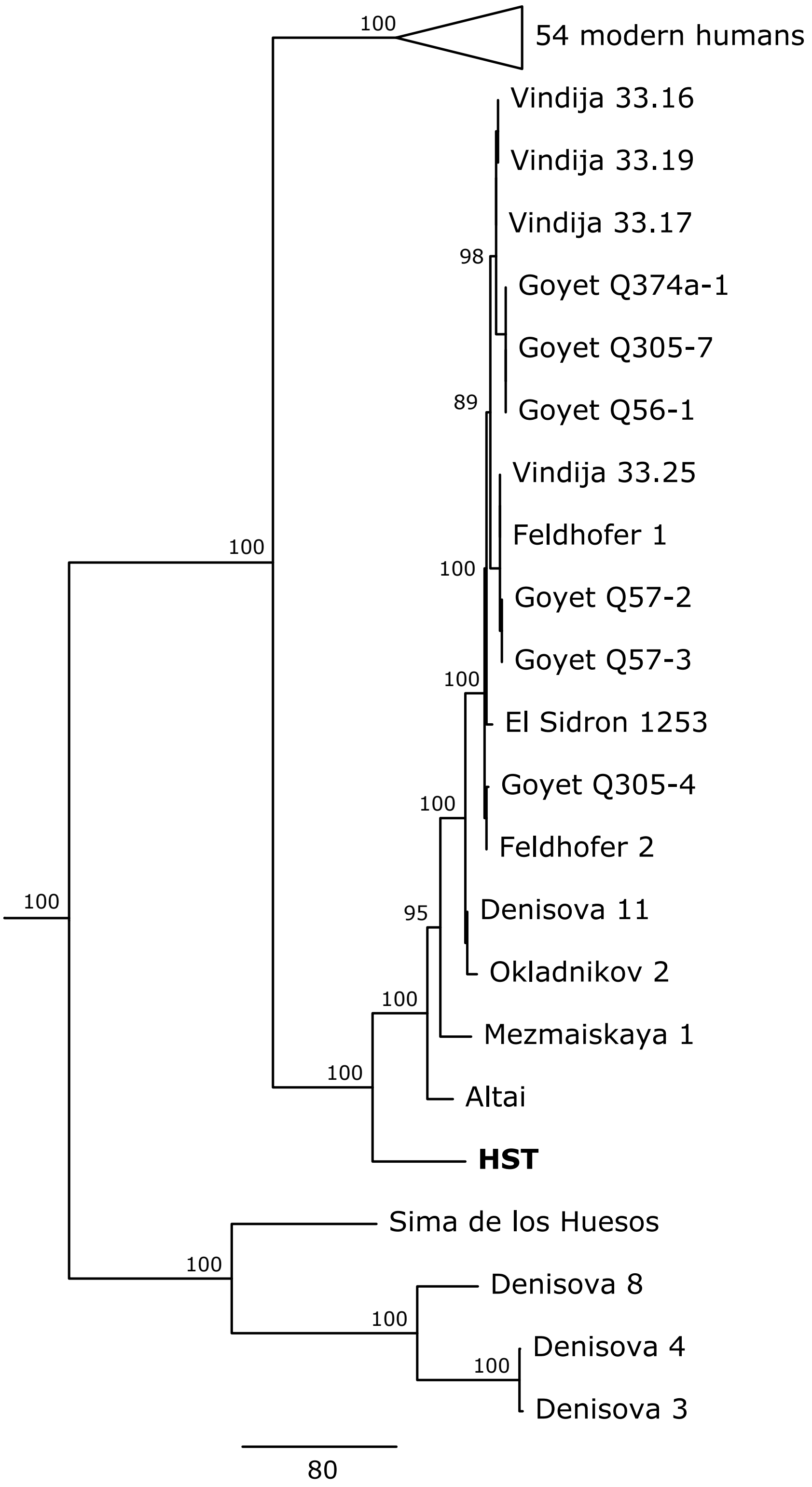

Supplementary Figure 5. Maximum parsimony tree built in MEGA6 with the complete mtDNA sequences (including D-loop) of: HST, 54 modern human, 17 Neanderthals, three Denisovans and Sima de los Huesos. The tree was tested with 1000 bootstrap iterations and 97% partial deletion and rooted with a chimpanzee mtDNA (not shown).

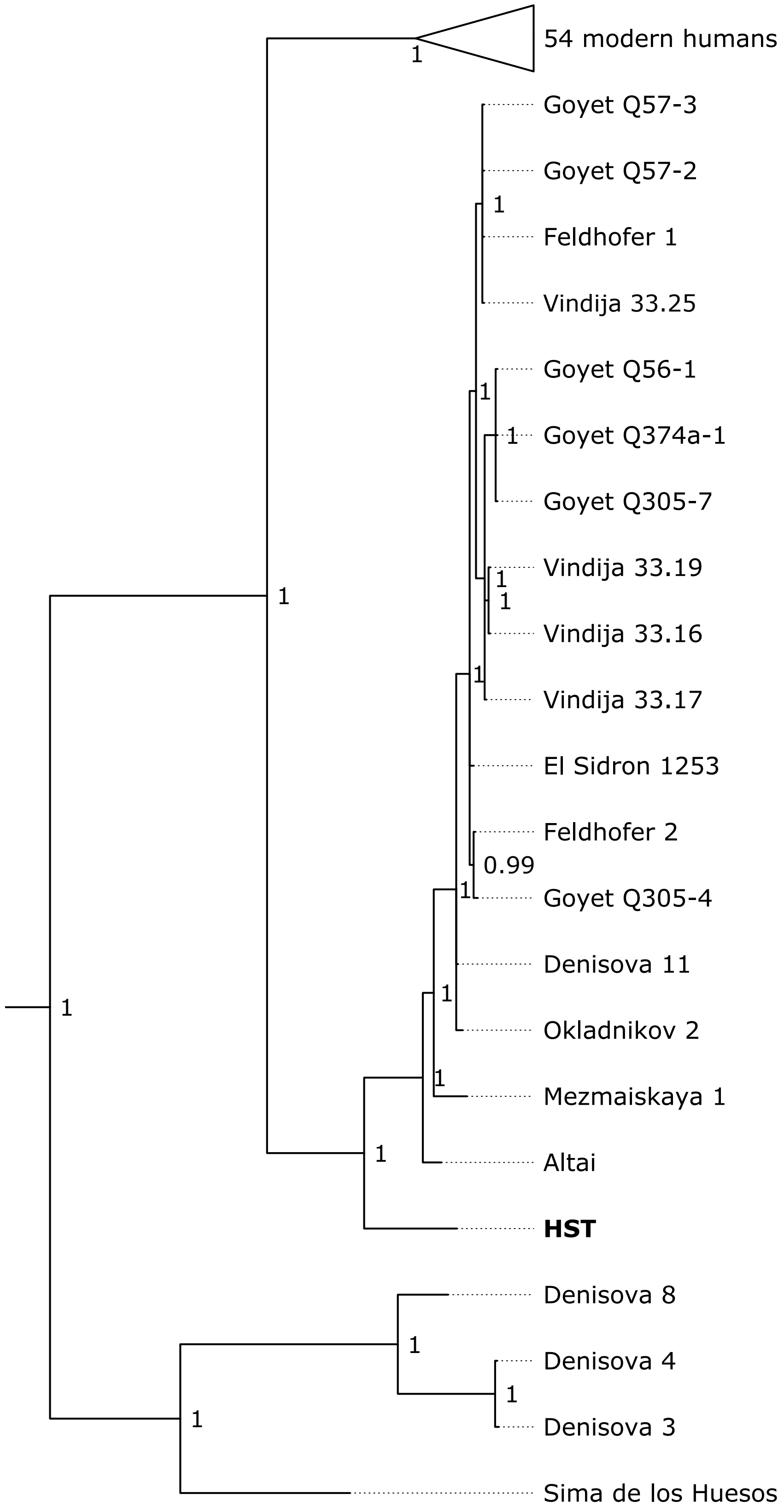

Supplementary Figure 6. Bayesian tree built in MrBayes with the coding mtDNA region of: HST, 54 modern human, 17 Neanderthals, three Denisovans and Sima de los Huesos. The tree was rooted with a chimpanzee mtDNA (not shown) and built including unassigned positions.

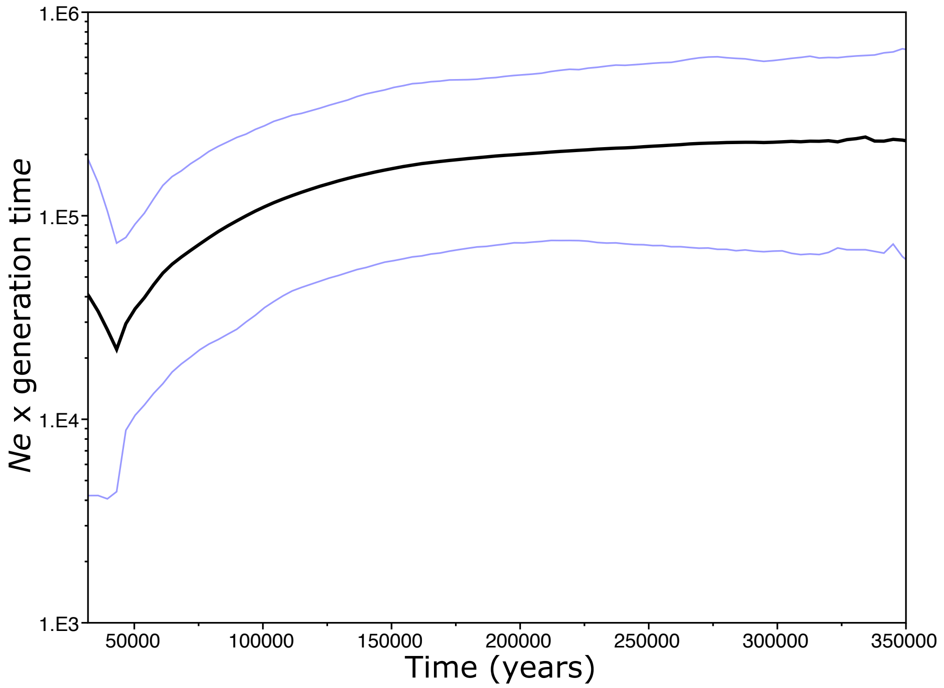

Supplementary Figure 7. Skyline plot depicting the Neanderthal mtDNA effective population size (N_e) x generation time (years) in logarithmic scale through time (from 350 ka to 32 ka). The black line represents the mean value while the purple lines the 95% HPD interval.

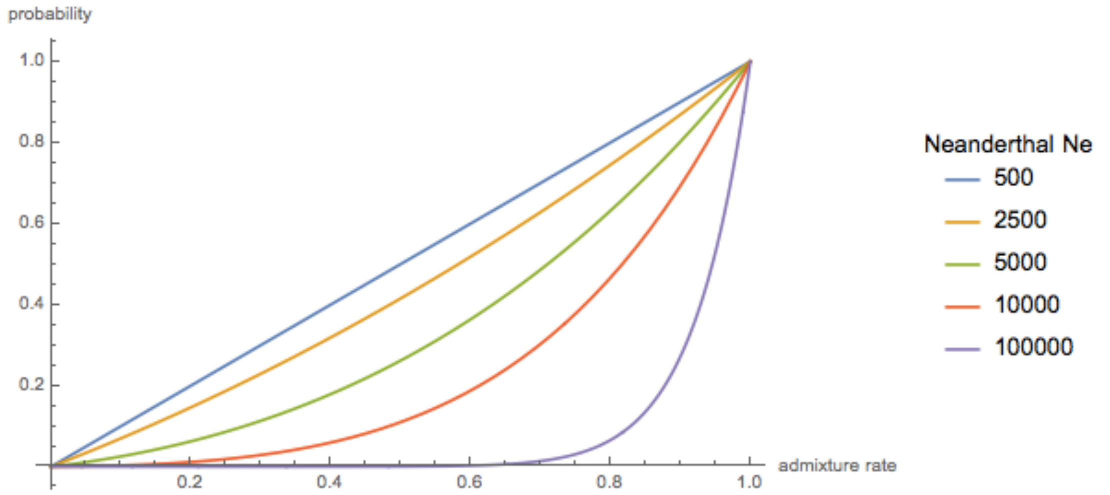

Supplementary Figure 8. Probability that all Neanderthal mitochondrial lineages originated from an admixture event with a BMH population, as a function of the admixture rate, for different values of the Neanderthal population size (N_e), here assumed constant.

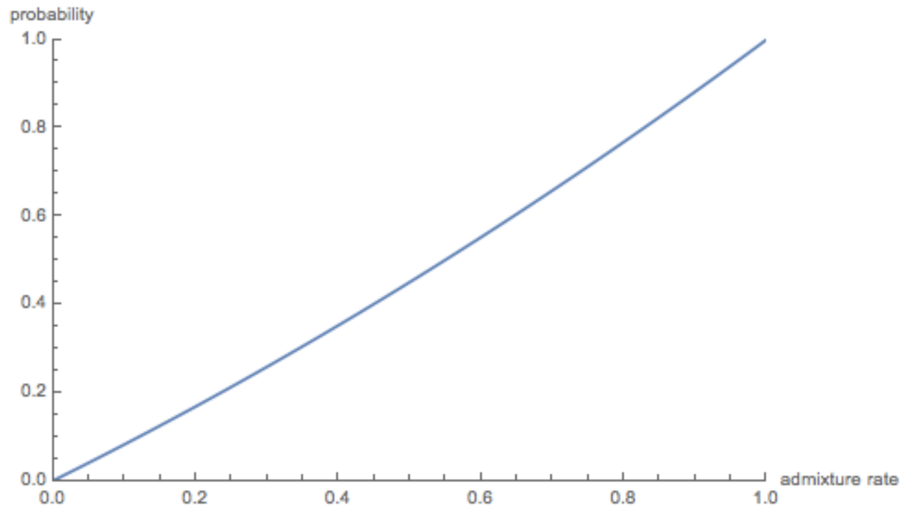

Supplementary Figure 9. Probability that all Neanderthal mitochondrial lineages originated from an admixture event with a BMH population, as a function of the admixture rate, for a piece-wise constant Neanderthal effective population size history that approximates the history estimated from the Bayesian skyline method.

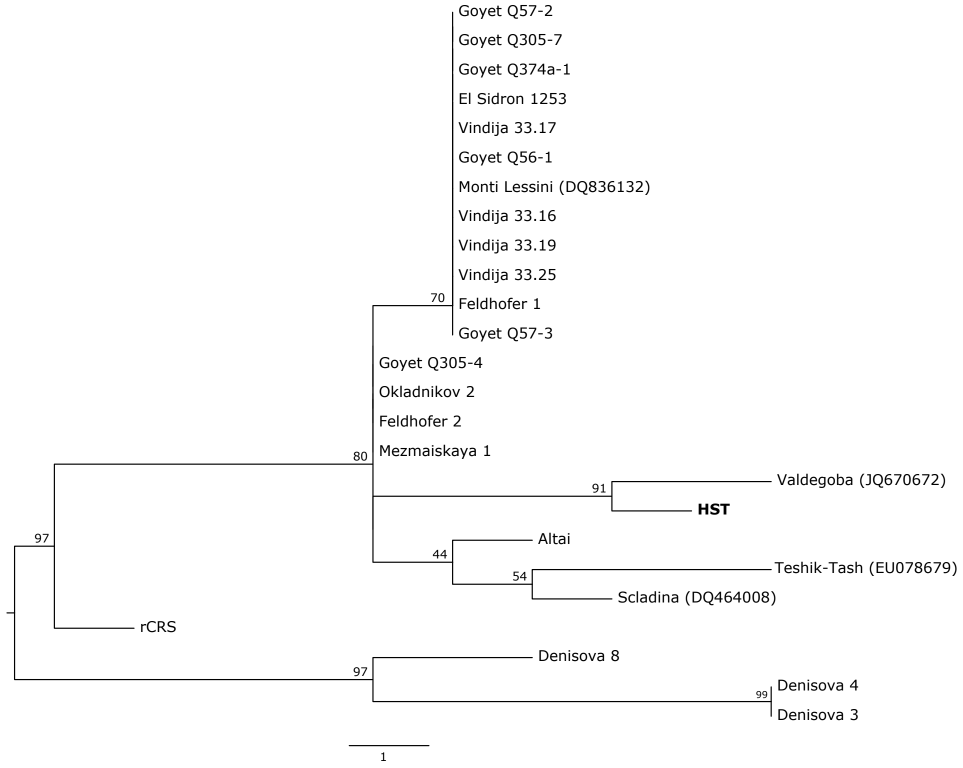

Supplementary Figure 10. Maximum parsimony tree built in MEGA6 with the HVRI regions of: HST, 20 Neanderthals, three Denisovans and rCRS. The tree was tested with 1000 bootstrap iterations and complete deletion. The accession numbers of the HVRI published sequences are reported in graphs next to individual name.

Supplementary Tables

Supplementary Table 1. Summary isotopic and dating results

Sample	Species	Element	%N-bone	%C-bone	%Sbone	Amount collagen (mg)	Yield (mg/g)	%Ccoll	%Ncoll	C/N	d13C	d15N	ZooMS	14C date
HST17	Neanderthal	Femur	n/a	n/a	n/a	20.0	63.8	41.5	14.8	3.3	-20.2	9.2	n/a	30570 ± 190 (GrA-43925)
HST21	Cervus elaphus	Tibia	2.1	7.4	0.1	11.8	38.9	41.5	14.7	3.3	-22.2	4.8	n/a	46975±1000 (MAMS-23208)
HST26	Cervus elaphus	Pelvis	2.1	7.5	0.1	19.3	58.8	42.4	15.0	3.3	-22.1	5.6	Red deer	49000±1000 (MAMS-23209)

Supplementary Table 2. Result statistics for each of the four mtDNA reference sequences (RNRS, Feldhofer 1, RSRS and rCRS) including mapped reads before and after duplicate removal and mapping quality filtering, percentage of fragments mapping the mtDNA reference, average coverage, average length, deamination at molecule termini and modern human contamination estimated with *contDeam*[4].

Reference	Merged Reads	Mapped reads	Duplicate removal	Mapping quality filter	Target DNA (%)	Duplication factor	Average Coverage (fold)	Insert size (bp)	3' deamination (%)	5' deamination (%)	*contDeam* (%) contamination (low - high)
RNRS	1,295,805	56,364	13,148	12,848	4.35	4.29	34.24	44.15	50.88	50.32	10 (9 - 11)
Feldhofer1	1,295,805	56,257	13,121	12,801	4.34	4.29	34.08	44.1	50.86	50.1	9.5 (8.5 - 10.5)
RSRS	1,295,805	56,604	13,130	12,790	4.37	4.31	34.05	44.11	50.82	50.26	10.5 (9.5 - 11.5)
rCRS	1,295,805	56,699	13,105	12,750	4.37	4.33	33.92	44.07	50.74	50.42	10.5 (9.5 - 11.5)

Supplementary Table 3. Result statistics of shotgun sequencing and mapping against the hg19 reference genome, duplicate removal and mapping quality filtering, percentage of human reads, average length and deamination at molecule termini.

Library ID	Raw Reads	Merged Reads	Mapped reads	Duplicate removal and quality filter	Endogenous DNA (%)	Insert size (bp)	3' deamination (%)	5' deamination (%)
GA87	554,766	242,521	1,110	713	0.46	41.12	32	42.48

Supplementary Table 4. Watterson's estimator among Neanderthal mtDNAs with and without HST.

Group	# segregating sites	n (# individuals)	*θw*
Neanderthals without HST	78	17	1.37E-03
Neanderthals with HST	145	18	2.50E-03

Supplementary Table 5. Pairwise nucleotide distance calculated by the number of nucleotide differences of modern human, Denisovan, Sima del los Heusos and other Neanderthal mtDNAs to the HST mtDNA.

	Nucleotide distance to HST	
**MtDNA dataset**	**Average**	**Interval**
311 modern humans	201	187-214
17 Neanderthals	104	89-111
3 Denisovans	362	354-366
Sima de los Huesos	292	na

Supplementary Table 6. Eight Neanderthal mtDNAs with radiocarbon dates used in BEAST analyses. Radiocarbon dates were calibrated using Oxcal 4.2[5] reporting 95,4% confidence intervals in years before present (rounded to the nearest decade).

Sample	Initial value	Lower value	Upper value	^{14}C date uncalibrated (lab-#)	Publication radiocarbon date	Publication mtDNA
Felhofer 1	43,710	42,670	44,750	39,900±620 (ETH-20981)	Schmitz et al. 2002	Briggs et al. 2009
Felhofer 2	43,265	42,190	44,340	39,240±670 (ETH-19660)	Schmitz et al. 2002	Briggs et al. 2009
Vindija 33.16	43,710	39,240	48,180	38,310±2,130 (U-n/a)	Serre et al. 2004	Green et al. 2008
El Sidrón 1253	43,040	40,300	47,050	40,840±1,200 (Beta-192065)<br>37,300±830 (Beta-192066)<br>38,240±890 (Beta-192067)	Lalueza-Fox et al. 2005 (teeth and bone level III)	Briggs et al. 2009
Goyet Q56-1	42,540	42,080	43,000	38,440+340-300(GrA-46170)	Rougier et al. 2016	Rougier et al. 2016
Goyet Q57-2	41,210	40,620	41,800	36,590+300-270(GrA-54024)	Rougier et al. 2016	Rougier et al. 2016
Goyet Q57-3	42,430	41,960	42,900	38,260+350-310(GrA-60019)	Rougier et al. 2016	Rougier et al. 2016
Goyet Q305-4	44,290	43,430	45,150	40,690+480,400(GrA-46176)	Rougier et al. 2016	Rougier et al. 2016

Supplementary Table 7. Log Marginal Likelihoods for Stepping stone (SS) and Path Sampling (PS) evaluation for dating analyses (four models compared) and skyline plot reconstruction (two models compared). For each model three runs each of 50Ma states were combined after 10% burn-in.

**Dating analyses**		**Log marginal Likelihood**	
Tree prior	Clock	Stepping-stone Sampling	Path Sampling
Constant	Strict	-28,641	-28,641
Constant	Relaxed	-28,642	-28,642
Skyline	Strict	-28,609	-28,610
Skyline	Relaxed	-28,611	-28,611
**Skyline plot**		**Log marginal Likelihood**	
Skyline	Strict	-22,276	-22,277
Skyline	Relaxed	-22,273	-22,286

Supplementary Table 8. Time to fixation (in 1000 years, with 1gen=29 years) for various mtDNA N_e and introgressing proportions.

**Introgressing mtDNA proportion**	**Neanderthal N_e (mtDNA)**									
	**10**	**50**	**100**	**250**	**500**	**1000**	**2500**	**5000**	**7500**	**10000**
**1×10^{-5}**	1	3	6	14	29	58	145	290	435	580
**1×10^{-4}**	1	3	6	14	29	58	145	290	435	580
**1×10^{-3}**	1	3	6	14	29	58	145	290	435	580
**5×10^{-3}**	1	3	6	14	29	58	145	289	434	579
**0.01**	1	3	6	14	29	58	144	289	433	577
**0.02**	1	3	6	14	29	57	144	287	431	574
**0.05**	1	3	6	14	28	57	141	283	424	565
**0.1**	1	3	5	14	27	55	137	275	412	550
**0.15**	1	3	5	13	27	53	134	267	401	534
**0.2**	1	3	5	13	26	52	129	259	388	518

Supplementary Notes

Supplementary Note 1: Archaeology of Hohlenstein-Stadel and the archaic femur

The femur diaphysis was found on August 26th 1937 during a test excavation at the entrance-zone of Hohlenstein-Stadel (HST) cave, underneath the foundation of a wall, which blocked the access to the cave since the late 16th century. A museum's inventory (Hahn) lists the object under "Probegrabung unter Mauer, 4. Hieb, No. 3668" and the find-number was written directly onto the bone after the excavation. Because of its archaic morphology the excavators suggested already in their first publications that the finding was a skeletal element of a Neanderthal individual[6-8]. It was not until several decades later that the specimen was published and morphologically described in detail[9]. The absence of the bone's epiphyses, the presence of gnawing marks of large carnivores on both ends of the long bone[10], as well as the absence of other remains from archaic humans in the cave, suggest that the femur was brought inside Hohlenstein-Stadel by carnivores or modified by carnivores after the initial deposition of the skeleton.

The femur was discovered at the entrance of the cave in a horizon that was correlated with an inner layer of the cave, known as the "Black Mousterian", as described by the archaeologists during excavations in the 1930s. Inside the cave the "Black Mousterian" was found at the lowermost of the stratigraphic sequence associated with Middle Paleolithic artifacts. During recent excavations on the forecourt of the cave, a displaced layer of black sediment was discovered (layer BG)[11] and radiocarbon dated to >50,000 ^{14}C years BP (ETH-38795)[12]. This displaced horizon from the forecourt can be correlated with the layer of the "Black Mousterian" from inside the cave as well as with the horizon where the HST femur was found. In fact, layer BG was discovered only a few meters away from the spot where the hominin remain was unearthed and showed clear indication of periglacial movements of sediments from inside the cave to the forecourt. Is suggested that the black layer containing the HST femur was also not *in situ* but displaced from the deeper chamber of the cave. A substantial amount of microfauna was discovered in the forecourt BG horizon, suggesting moderate climatic conditions that were also confirmed by the presence of a limited number of arctic faunal species[11]. The composition of small mammals is unknown in southwestern Germany during the Marine Isotope Stage 3 (MIS 3), but it is also unlikely to have originated during the Eemian interglacial period (MIS 5e). Therefore it is more plausible that this layer formed during one of the moderate interstadial periods at the beginning of the last glaciation (MIS 5c or 5a)[9,13].

Supplementary Note 2: Isotopic results

Collagen extraction was performed at Tübingen University and followed the method from Longin[14] and described in Bocherens et al[15]. Isotopic measurements of $\delta^{15}N$ and $\delta^{13}C$ were done using an elemental analyzer NC2500 connected to a Thermo Quest Delta + XL isotopic ratio mass spectrometer. The isotopic ratios are expressed using the "δ" (delta) value as follows: $\delta^{13}C = [(^{13}C/^{12}C)_{sample}/(^{13}C/^{12}C)_{reference}-1] \times 1000‰$, $\delta^{15}N = [(^{15}N/^{14}N)_{sample}/(^{15}N/^{14}N)_{reference}-1] \times 1000‰$. The standard for $\delta^{13}C$ is the internationally defined marine carbonate V-PDB. For $\delta^{15}N$ the atmospheric nitrogen (AIR) is used. Analytical error based on laboratory standards is ±0.1‰ for $\delta^{13}C$

values and ±0.2‰ for $\delta^{15}N$. The chemical preservation of collagen is expressed through the atomic ratio of $C_{coll}:N_{coll}$, whose acceptable range of variation is 2.9–3.6[16], while the nitrogen content (N_{coll}) should be above 5%[17].
The attempt to directly radiocarbon date HST femur resulted in an age of 34,130-34,880 years cal BP (GrA-43925: 30,570 ± 190 ^{14}C years). This date is inconsistent with the estimated end of the Mousterian around 40 ka[18] and with the femur's stratigraphic position (see Supplementary Note 1). Stable isotopic composition in the collagen of HST femur (HST 17) differed from that of late Neanderthals[1] (Supplementary Fig. S1 and Table S1). In fact, the lower $\delta^{13}C$ and $\delta^{15}N$ values in the hominin specimen correspond to a different ecology from the one of late Neanderthals in western-central Europe. We can exclude contamination as the responsible factor for the obtained stable isotope values. While few percent of modern carbon could explain the measured radiocarbon date, this would not affect notably the isotopic composition. Furthermore, we analyzed two faunal remains (HST 21 and HST 26) from the same stratigraphic unit where the HST femur was discovered. Collagen from both specimens was extracted and radiocarbon dated to >49,000 ^{14}C years (HST 26) and 46,975 ± 1000 ^{14}C years (HST 21) (Table S1). In this time range, both dates can be considered beyond the limit of radiochronometric dating, where a minimal proportion of contamination with modern carbon could result in a wrongly finite date. Both specimens were initially identified as red deer on a morphological basis and ZooMS analyses[19] performed on HST 26 collagen confirmed the species assignment. Moreover, collagen of both cervid remains provided $\delta^{13}C$ isotopic values distinctively lower than individuals of the same species grazing in open steppic habitats[20] (Supplementary Table S1, Supplementary Fig. S2). Isotopic evidence thus suggests that both the hominin and the two deer from Hohlenstein-Stadel Black Mousterian lived in a more forested rather than a steppic environment typical for the Late Neanderthals in northwestern Europe.

Supplementary Note 3: Reference mapping bias

In order to evaluate the impact in the consensus reconstruction of the four different mapping references, we used the software MUSCLE[21] to first generate a multiple genome alignment of the four consensus with 17 Neanderthal mtDNAs[22-27] and rCRS[28] as out-group. We excluded the individual Goyet Q57-1 from Rougier et al.[27] in the phylogeny and further analyses because around 2% of mtDNA positions were unassigned. We then built a phylogenetic tree with the maximum parsimony method (SPR algorithm) in MEGA6[29]. A total of 16,255 positions were considered in the phylogeny with complete deletion and 1000 replicates as bootstrap support (Supplementary Fig. S4A). Looking at the tree topology, all four consensus variants are placed on a basal mtDNA Neanderthal lineage but with different branch lengths. In particular, when the two modern human mtDNA sequences (rCRS and RSRS) are used as mapping references, the resulting consensus sequences show shorter phylogenetic branches. We interpret this phenomenon as a reference bias that facilitates contaminant reads to map better than the endogenous ones, producing consensus sequences closer to the modern human references. Instead, when using Neanderthal references (Feldhofer 1 and RNRS) that are phylogenetically more similar to the endogenous mtDNA we observe a greater amount of derived positions. After visual inspection (Methods section) we could confirm that all derived positions observed in the RNRS consensus are indeed of endogenous origin and thus this sequence was used for phylogenetic and mtDNA diversity analyses. We further

observed that 18 of the 19 inconsistent positions across the four consensus sequences are placed in the D-loop (rCRS pos. 16023-577) where the most polymorphic regions of the mtDNA are located (HVRI and HVRII). We then removed the D-loop from the alignment and constructed an additional maximum parsimony tree with the same parameters described above (15,345 positions) (Supplementary Fig. S4B). As expected, the reference bias highlighted previously was overcome. Thus we used the most conservative coding region consensus to perform BEAST and additional phylogenetic analyses.

Supplementary Note 4: Mutation rate mtDNA

The enlarged dataset of 18 complete Neanderthal mtDNAs provide us with the opportunity to create for the first time to our knowledge a skyline plot for Neanderthal population assuming panmixia. We used only the mtDNA coding region and not the whole molecule for BEAST analyses because the vast majority of unassigned nucleotides in the published Neanderthal mtDNAs are located in the D-loop. This region is the most variable of the mtDNA with the highest mutation rate. Having the D-loop poorly covered but using a mutation rate for the whole molecule would effectively accelerate the mutation rate of the coding region. Therefore as molecular clock we set a fixed rate of $1.57*10^{-8}$ μ / site / year[30] calculated for the coding region of modern humans with ancient mtDNAs as calibration points. Additionally we used the eight radiocarbon Neanderthal dates (Supplementary Table S6) as time anchors on the Neanderthal branch. When we tried to estimate the mutation rate independently with these dates as tip calibrations, BEAST runs did not converge or provided a non-realistic rate of one order of magnitude lower than observed for modern humans. Two possible reasons could explain these patterns. First, late Neanderthals ages are at the limit for the radiocarbon dating method, therefore minimal contaminations with modern collagen could result in considerably younger ages, as reported for El Sidron Neanderthal specimens[31]. Second, the eight radiocarbon dated Neanderthals have an average age in a range of only 3,000 years (mean values from ~41 to ~44 ka) (Supplementary Table S6). Therefore there might be not enough temporal depth to calibrate the molecular clock in a phylogenetic tree with divergence times in the order of several hundred thousands of years. For BEAST analyses we thus assumed that the mtDNA mutation rate of Neanderthals would be similar to the one of modern humans.

Supplementary Note 5: Likelihood of basal modern human-Neanderthal mtDNA admixture rate

We are interested in obtaining the probability that all Neanderthal mtDNAs are transferred from a branch that splits off basal from the modern human (BMH) lineage, assuming an instantaneous admixture event at some point in the past, before modern human populations started diverging into present-day groups. This is effectively a likelihood of our data given the rate of this admixture event. Let g(n,j,t) be the probability of there being j lineages at time t in the past, given that there were n lineages at time 0 in the present, measuring time in coalescent units[32]:

$$g(n,j,t) = \sum_{k=j}^{n} e^{-\frac{k(k-1)t}{2}} \frac{(2k-1)(-1)^{k-j} j_{(k-1)} n_{[k]}}{j!\,(k-j)!\,n_{(k)}}$$

where

$$a_{(x)} = a(a+1)\dots(a+x-1)$$
$$a_{[x]} = a(a-1)\dots(a-x+1)$$
$$a_{(0)} = a_{[0]} = 1$$

Also, let b(j,n,p) be the binomial probability of j successes out of n trials, when p is the probability of success:

$$b(j,n,p) = \binom{n}{j} p^j (1-p)^{n-j}$$

At the time of admixture, we can treat each lineage transferred (backwards in the past) from BMH to the Neanderthal population as a success with probability equal to the admixture rate, assuming the admixture event was unidirectional. Then, for a given admixture rate r and a number y of Neanderthal mitochondrial lineages sampled in the present, the probability that all Neanderthal mitochondrial lineages originally came from the BMH population via this admixture event is:

$$P[y,r,t] = \sum_{j=1}^{y} g(y,j,t)\, b(j,j,r)$$

where the sum is over the possible number of Neanderthal lineages that may have existed at time t in the past.

The time t is measured in coalescent units. Assuming constant population size, this is equal to the time τ in generations scaled by twice the effective population size (N_e):

$$t = \frac{\tau}{2N_e}$$

For our analysis, we assume 29 years per generation, and set y = 18, as this is the number of Neanderthal mitochondrial genomes sampled so far. We begin by assuming the admixture event happened 300,000 years more anciently than the time of sampling. Assuming the sampling occurred 40,000 years ago (age of the latest Neanderthal mtDNAs), the event is therefore set to be more anciently than the deepest modern human population splits but more recently than the Neanderthal-Denisovan population split[25]. Using Mathematica[33], we can plot the probability P[y,r,t] as a function of the admixture rate r, for various constant Neanderthal effective population sizes (Supplementary Fig. S8).
We can see that, predictably, P[y,r,t] is an increasing function of the admixture rate r. For low Neanderthal N_e, this probability is almost exactly equal to the admixture rate: when N_e is low, by the time the admixture event happens (going backwards into the past) there is only a single lineage in the Neanderthal population with high probability. The probability that this single lineage enters the Neanderthal population is Bernoulli-distributed with parameter r. For larger Neanderthal N_e, there is some non-trivial probability that two or more lineages make it all the way to the time in the past when the admixture event happened. This probability is smaller the larger the N_e.

In a diploid constant randomly-mating population with equal number of males and females and non-overlapping generations, the mitochondrial N_e is expected to be a quarter of the autosomal N_e[34]. Assuming the Neanderthal mitochondrial N_e was a quarter of the estimated autosomal N_e (2,000)[35], this probability is well approximated by the blue line in Supplementary Figure 8.

We can also use the compound (generation time * N_e) estimates from the Bayesian skyline method (Supplementary Fig. S7), assuming 29 years per generation, as a proxy for the history of mitochondrial population size changes in Neanderthals. To make computations feasible, we split this history into a piece-wise constant history with 3 periods. Going from the time of sampling into the past, these were set to be:

Period 1: 10,000 years with $2N_e = (40{,}000 / 29)$

Period 2: 20,000 years with $2N_e = (20{,}000 / 29)$

Period 3: 270,000 years with $2N_e = (200{,}000 / 29)$

Under these conditions, P[y,r,t] is almost a linear function of the admixture rate, suggesting a very high admixture rate may not be required for all descendant lineages to have originated from this event (Supplementary Fig. S9).

Supplementary Information References

1 Wißing, C. *et al.* Isotopic evidence for dietary ecology of late Neandertals in North-Western Europe. *Quaternary International* **411**, 327-345 (2016).

2 Beauval, C., Lacrampe-Cuyaubere, F., Maureille, B. & Trinkaus, E. Direct radiocarbon dating and stable isotopes of the neandertal femur from Les Rochers-de-Villeneuve (Lussac-les-Châteaux, Vienne). *Bulletins et mémoires de la Société d'Anthropologie de Paris* **18**, 35-42 (2006).

3 Bocherens, H., Drucker, D. G., Billiou, D., Patou-Mathis, M. & Vandermeersch, B. Isotopic evidence for diet and subsistence pattern of the Saint-Cesaire I Neanderthal: review and use of a multi-source mixing model. *J Hum Evol* **49**, 71-87, doi:10.1016/j.jhevol.2005.03.003 (2005).

4 Renaud, G., Slon, V., Duggan, A. T. & Kelso, J. Schmutzi: estimation of contamination and endogenous mitochondrial consensus calling for ancient DNA. *Genome Biol* **16**, 224, doi:10.1186/s13059-015-0776-0 (2015).

5 Ramsey, C. B. & Lee, S. Recent and Planned Developments of the Program Oxcal. *Radiocarbon* **55**, 720-730 (2013).

6 Völzing, O. Die Grabungen 1937 am Hohlestein im Lonetal. *Fundberichte aus Schwaben* **NF 9**, 1-7 (1938).

7 Wetzel, R. Die Kopfbestattung und die Knochentrümmerstätte des Hohlensteins im Rahmen der Urgeschichte des Lonetals. *Verhandl. Dt. Ges. f. Rassenforschung* **9**, 193-212 (1938).

8 Wetzel, R. Der Hohlestein im Lonetal. Dokumente alteuropäischer Kulturen vom Eiszeitalter bis zur Völkerwanderung. *Mitteilungen des Vereins für Naturwissenschaft und Mathematik in Ulm (Donau)* **27**, 21-75 (1961).

9 Kunter, M. & Wahl, J. Das Femurfragment eines Neandertalers aus der Stadelhöhle des Hohlensteins im Lonetal. *Fundberichte aus Baden-Württemberg* **17**, 111-124 (1992).

10 Camarós, E., Münzel, S. C., Cueto, M., Rivals, F. & Conard, N. J. The evolution of Paleolithic hominin–carnivore interaction written in teeth: Stories from the Swabian Jura (Germany). *Journal of Archaeological Science: Reports* **6**, 798-809, doi:10.1016/j.jasrep.2015.11.010 (2016).

11 Jahnke, T. *Vor der Höhle. Zur Fundplatzgenese am Vorplatz des Hohlenstein-Stadel (Lonetal)* MA thesis thesis, Universität Tübingen, (2015).

12 Kind, C. J. *Löwenmensch und mehr. Die Ausgrabungen 2008 - 2013 in der Stadel-Höhle im Hohlenstein (Lonetal), Gemeinde Asselfingen, Alb-Donau-Kreis*. (in prep.).

13 Street, M., Terberger, T. & Orschiedt, J. A critical review of the German Paleolithic hominin record. *J Hum Evol* **51**, 551-579, doi:10.1016/j.jhevol.2006.04.014 (2006).

14 Longin, R. New method of collagen extraction for radiocarbon dating. *Nature* **230**, 241-242 (1971).

15 Bocherens, H. *et al.* Paleobiological implications of the isotopic signatures (C-13, N-15) of fossil mammal collagen in Scladina cave (Sclayn, Belgium). *Quaternary Research* **48**, 370-380, doi:DOI 10.1006/qres.1997.1927 (1997).

16 Deniro, M. J. Postmortem Preservation and Alteration of Invivo Bone-Collagen Isotope Ratios in Relation to Paleodietary Reconstruction. *Nature* **317**, 806-809, doi:DOI 10.1038/317806a0 (1985).
17 Ambrose, S. H. Preparation and Characterization of Bone and Tooth Collagen for Isotopic Analysis. *Journal of Archaeological Science* **17**, 431-451, doi:Doi 10.1016/0305-4403(90)90007-R (1990).
18 Higham, T. *et al.* The timing and spatiotemporal patterning of Neanderthal disappearance. *Nature* **512**, 306-309, doi:10.1038/nature13621 (2014).
19 Buckley, M., Collins, M., Thomas-Oates, J. & Wilson, J. C. Species identification by analysis of bone collagen using matrix-assisted laser desorption/ionisation time-of-flight mass spectrometry. *Rapid Commun Mass Spectrom* **23**, 3843-3854, doi:10.1002/rcm.4316 (2009).
20 Bocherens, H., Drucker, D. G. & Madelaine, S. Evidence for a (15)N positive excursion in terrestrial foodwebs at the Middle to Upper Palaeolithic transition in south-western France: Implications for early modern human palaeodiet and palaeoenvironment. *J Hum Evol* **69**, 31-43, doi:10.1016/j.jhevol.2013.12.015 (2014).
21 Edgar, R. C. MUSCLE: multiple sequence alignment with high accuracy and high throughput. *Nucleic Acids Res* **32**, 1792-1797, doi:10.1093/nar/gkh340 (2004).
22 Briggs, A. W. *et al.* Targeted retrieval and analysis of five Neandertal mtDNA genomes. *Science* **325**, 318-321, doi:10.1126/science.1174462 (2009).
23 Green, R. E. *et al.* A complete Neandertal mitochondrial genome sequence determined by high-throughput sequencing. *Cell* **134**, 416-426, doi:10.1016/j.cell.2008.06.021 (2008).
24 Skoglund, P. *et al.* Separating endogenous ancient DNA from modern day contamination in a Siberian Neandertal. *Proc Natl Acad Sci U S A* **111**, 2229-2234, doi:10.1073/pnas.1318934111 (2014).
25 Prufer, K. *et al.* The complete genome sequence of a Neanderthal from the Altai Mountains. *Nature* **505**, 43-49, doi:10.1038/nature12886 (2014).
26 Gansauge, M. T. & Meyer, M. Selective enrichment of damaged DNA molecules for ancient genome sequencing. *Genome Res* **24**, 1543-1549, doi:10.1101/gr.174201.114 (2014).
27 Rougier, H. *et al.* Neandertal cannibalism and Neandertal bones used as tools in Northern Europe. *Sci Rep* **6**, 29005, doi:10.1038/srep29005 (2016).
28 Andrews, R. M. *et al.* Reanalysis and revision of the Cambridge reference sequence for human mitochondrial DNA. *Nat Genet* **23**, 147, doi:10.1038/13779 (1999).
29 Tamura, K., Stecher, G., Peterson, D., Filipski, A. & Kumar, S. MEGA6: Molecular Evolutionary Genetics Analysis version 6.0. *Mol Biol Evol* **30**, 2725-2729, doi:10.1093/molbev/mst197 (2013).
30 Fu, Q. *et al.* A revised timescale for human evolution based on ancient mitochondrial genomes. *Curr Biol* **23**, 553-559, doi:10.1016/j.cub.2013.02.044 (2013).
31 Wood, R. E. *et al.* A New Date for the Neanderthals from El Sidron Cave (Asturias, Northern Spain)*. *Archaeometry* **55**, 148-158, doi:10.1111/j.1475-4754.2012.00671.x (2013).

32 Tavaré, S. Line-of-descent and genealogical processes, and their applications in population genetics models. *Theoretical population biology* **26**, 119-164 (1984).
33 Mathematica v. 11.0 (Champaign, IL, 2016).
34 Charlesworth, B. Fundamental concepts in genetics: effective population size and patterns of molecular evolution and variation. *Nat Rev Genet* **10**, 195-205, doi:10.1038/nrg2526 (2009).
35 Kuhlwilm, M. *et al.* Ancient gene flow from early modern humans into Eastern Neanderthals. *Nature* **530**, 429-433, doi:10.1038/nature16544 (2016).
